# Value-based preoperative assessment in a large academic hospital

**DOI:** 10.1186/s44158-024-00161-7

**Published:** 2024-07-08

**Authors:** Maurizio Cecconi, Giulia Goretti, Andrea Pradella, Patrizia Meroni, Martina Pisarra, Guido Torzilli, Marco Montorsi, Antonino Spinelli, Alessandro Zerbi, Carlo Castoro, Paolo Casale, Efrem Civilini, Vittorio Quagliuolo, Marco Klinger, Giuseppe Spriano, Domenico Vitobello, Leonardo Maradei, Bernhard Reimers, Federico Piccioni, Maria Rosaria Martucci, Niccolò Stomeo, Elena Vanni, Marco Babbini, Roberta Monzani, Maria Rosaria Capogreco, Michele Lagioia, Massimiliano Greco

**Affiliations:** 1 Department of Biomedical Sciences, Humanitas University, Pieve Emanuele, Milan, Italy; 2 Department of Anaesthesiology and Intensive Care, IRCCS Humanitas Research Hospital, Rozzano, Milan Italy; 3 IRCCS Humanitas Research Hospital, Rozzano, Milan Italy; 4 Department of Economics, Management and Quantitative Methods, University of Milan, Milan, 20122 Italy; 5 Department of Surgery, IRCCS Humanitas Research Hospital, Rozzano, Milan Italy; 6 Department of Cardiology, Humanitas Clinical and Research Center IRCCS, Milan, Italy

**Keywords:** Outcomes, Patient-centred care, Patient empowerment, Preoperative evaluation, Preoperative assessment, Quality, Risk, Surgery, Value-based healthcare

## Abstract

**Background:**

Value-based healthcare (VBHC) is an approach that focuses on delivering the highest possible value for patients while driving cost efficiency in health services. It emphasizes improving patient outcomes and experiences while optimizing the use of resources, shifting the healthcare system’s focus from the volume of services to the value delivered. Our study assessed the effectiveness of implementing a VBHC-principled, tailored preoperative evaluation in enhancing patient care and outcomes, as well as reducing healthcare costs.

**Methods:**

We employed a quality improvement, before-and-after approach to assessing the effects of implementing VBHC strategies on the restructuring of the preoperative evaluation clinics at Humanitas Research Hospital. The intervention introduced a VBHC-tailored risk matrix during the postintervention phase (year 2021), and the results were compared with those of the preintervention phase (2019). The primary study outcome was the difference in the number of preoperative tests and visits at baseline and after the VBHC approach. Secondary outcomes were patient outcomes and costs.

**Results:**

A total of 9722 patients were included: 5242 during 2019 (baseline) and 4,480 during 2021 (VBHC approach). The median age of the population was 63 (IQR 51–72), 23% of patients were classified as ASA 3 and 4, and 26.8% (2,955 cases) were day surgery cases. We found a considerable decrease in the number of preoperative tests ordered for each patient [6.2 (2.5) vs 5.3 (2.6) tests, *p* < 0.001]. The number of preoperative chest X-ray, electrocardiogram, and cardiac exams decreased significantly with VBHC. The length of the preoperative evaluation was significantly shorter with VBHC [373 (136) vs 290 (157) min, *p* < 0.001]. Cost analysis demonstrated a significant reduction in costs, while there was no difference in clinical outcomes.

**Conclusions:**

We demonstrated the feasibility, safety, and cost-effectiveness of a tailored approach for preoperative evaluation. The implementation of VBHC enhanced value, as evidenced by decreased patient time in preoperative evaluation and by a reduction in unnecessary preoperative tests.

**Supplementary Information:**

The online version contains supplementary material available at 10.1186/s44158-024-00161-7.

## Background

Preoperative evaluation is essential for patient safety and involves several members of the multidisciplinary team, including surgeons, anesthesiologists, and other healthcare providers, to identify organ dysfunction and stratify patient risk [1]. It is recognized as a central aspect of perioperative care, even if its effectiveness in terms of measurable healthcare outcomes is debated [2]. In recent years, the healthcare system has faced escalating pressures and expectations, driven by a growing population of frail and elderly surgical patients requiring more intensive perioperative care. This challenge is compounded by financial constraints and rising healthcare costs, which constrain the range of available healthcare options [3]. As a result, even if an effective preoperative assessment is necessary to reduce preventable outcomes during surgical procedures, several concerns are frequently raised about its efficiency, including (i) the overlap and redundancy between surgical staging visits and preoperative assessment visits, (ii) waste and inefficiencies for patients and providers (i.e., multiple hospital accesses for different visits and tests, repeated testing over time) [4], and (iii) the increased workload for healthcare professionals due to inefficient processes [5].

Diverse approaches have been used by healthcare organizations [6]. Value-based healthcare (VBHC) has been proposed as an innovative framework for transforming healthcare systems [7], along with a strategic agenda for moving to a high-value healthcare delivery system [8]. The objective and innovation of VBHC is to establish the value that matters to patients as opposed to expanding service volume [9–12]. In this view, better health outcomes are reflected by increasing both the health status and patients’ and other stakeholders’ values.

While there is emerging evidence of the benefits of VBHC [13], studies have considered VBHC deployment as a means of enhancing preoperative clinics [14]. Our group recently published a report on the application of the VBHC system to a single surgical unit [15], but no studies have examined the effects of preoperative VBHC interventions at the hospital level.

Consequently, the aim of this study was to assess the clinical and organizational impacts of revising the preoperative pathway across multiple units of a large surgical hospital, aligning it with the principles of VBHC.

## Methods

We conducted a before-and-after study on quality improvement in Humanitas Research Hospital, an academic hospital in Milan with a large surgical volume. The study received IRB approval No. 44/21, September 2021, from the Humanitas Research Hospital Independent Ethical Committee. Patient informed consent was waived due to the anonymization of the data, gathered from the Electronic Health Record (EHR) system. Inclusion criteria were access to preoperative evaluation clinics in 2019 and 2021, for the units involved in the VBHC initiative. We defined the primary outcome as the absolute variation in the number of preoperative tests performed per patient before the VBHC intervention (year 2019, baseline data) and after the VBCH intervention (year 2021). The year 2020 was used to train the personnel. Therefore, the study lasted 3 years in total.

Secondary study endpoints were identified by observing the standard VBHC 3-tiered hierarchy of outcomes: [7]

i) Clinical outcome: postoperative mortality
ii) Process impacts: ICU admission, request for chest X-rays and cardiac visits, lead time (pathway’s total time), and value time (i.e., the time spent in value-added activities)
iii) Sustainability impact: tests/consults requested after preoperative assessment, and surgical cancellation due to inadequate preoperative assessment

Finally, an analysis of the economic resources saved during the initiative was also planned, for each VBHC initiative, and the results were divided into a three-level evaluation for analysis.

i) Revenues improvements
ii) Cost reduction
iii) Released resources

This study was performed in subsequent phases:

1. *Baseline phase*: prior to the VBHC initiative, the hospital protocol for preoperative risk assessment was based on administering a uniform set of baseline tests across all surgical units, with additional tests according to patient age. Typically, patients would undergo these standardized tests at the beginning of the process before their medical evaluation, after which anesthesiologists or surgeons might request further assessments based on their professional judgment. This often led to redundant evaluations, inefficiencies, and a waste of both time and resources.

2. *Development of VBHC Initiative*: to address the discrepancy between the need for expert final evaluation and the aim to minimize unnecessary testing, we designed an intervention based on a tailored preoperative approach, and evaluated its efficacy according to VBHC principles.

Guided by a core team, we established 11 multidisciplinary teams Integrated Practice Units, composed of anaesthesiologists, surgeons, cardiologists, hospital operations managers, and healthcare quality experts. We established dedicated Integrated Practice Units (IPU) for each surgical specialty, inspired by the framework proposed in the 2016 National Institute of Clinical Excellence (NICE) guidelines [16]. Each IPU created a specific risk matrix based on two variables: (i) the patient’s medical risk, and (ii) the severity or invasiveness of the surgery.

For optimal effectiveness, the risk matrix needs to be straightforward and rapid to implement. It should replace the uniform, standard protocol of initial testing conducted at the outset of the preoperative evaluation process.

**Fig. 1:**
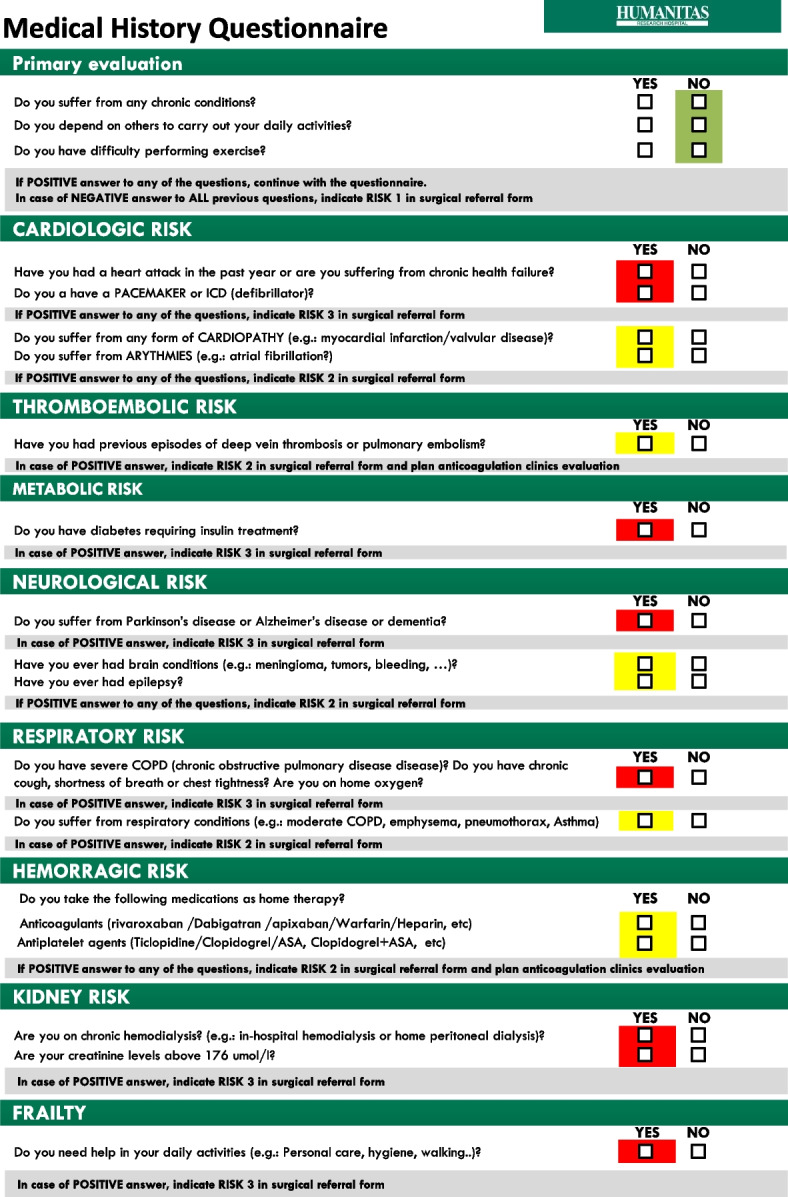
Medical risk anamnestic questionnaire

For the medical risk, we developed a standardized anamnestic questionnaire (Fig. 1) [17]. The medical risk questionnaire was designed to be straightforward to complete during the initial surgical consultation.

The proposing surgeon should also indicate surgical intervention risk. To standardize this process each IPU categorized all the surgical procedures they performed in categories of low, medium, and high surgical risk according to surgical invasiveness.

3. *The medical questionnaire validation phase* required a dedicated 3-month validation period because it was designed for use in this unique context, with two goals in mind: simplicity and effectiveness. As reported in (Supplemental material 2) the questionnaire follows a simple IF–THEN algorithm divided into 9 color-guided steps and can be completed in a few minutes based on patients’ answers.

**Fig. 2:**
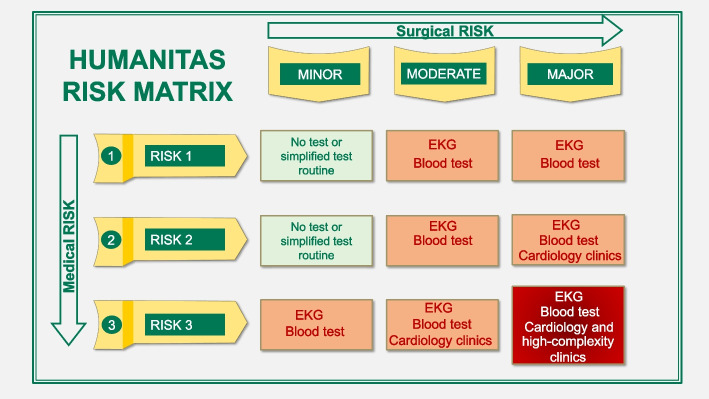
Humanitas pre-operative risk assessment matrix

Following the assignment of medical and surgical risks, these were aligned within a matrix comprising nine distinct strata, created by cross-referencing three levels of medical risk with three levels of surgical risk (Fig. 2). Each IPU decided the most appropriate preoperative tests for each stratum of the matrix, to create a specific preoperative pathway for each stratum and each IPUs.

In total, we developed eleven matrices (one for each IPU) for a total of 99 strata (9 for each of the 11 matrices). These 99 risk strata constituted the core of the personalized approach to perioperative risk, as each patient was included in one of the strata immediately on the day of the surgical proposal, well before the start of the preoperative evaluation process. Given the similarities between different strata, to simplify the process we ultimately synthesized the developed risk stratification in 40 different strata, divided into 11 IPUs (low, medium, and high complexity procedures).

The VBHC intervention was designed to comprehend the whole *cycle of patient care*, and to consider the reorganization of the process from the first visit of surgical proposal to the postoperative recovery. Within the developed VBHC approach the surgeon identifies the patient’s risk through the medical risk questionnaire and the surgery’s severity, and provides a final risk value from the matrix. The visits and medical tests for the pre-operative assessment are identified according to the matrix final risk value. For simplicity and synthesis, in each IPU’s matrix there are three levels of complexity, even if some can have more than 3 levels:

- *▪ Low complexity* includes surgeries classified as “minor severity” by surgeons and with medical risk class 1 and 2 (no test or simplified test routine)
- *▪ Medium complexity* includes surgeries classified as “intermediate severity” or “major severity” and with medical risk classes 1 and 2, or surgeries classified as “minor severity” or “intermediate severity” and with medical risk class 3 (blood test and ECG in addition to vital signs detection). For more invasive surgeries (“major severity surgeries”) classified as medical risk class 2, as well as intermediate severity surgeries under medical risk class 3, we incorporated an additional preoperative cardiology consultation.
- *▪ High complexity* includes “major severity” surgeries with medical risk class 3. We require a complete preoperative assessment with blood tests, an EKG, vital signs detection, a cardiac visit, and a multidisciplinary clinic dedicated to high-complexity patients.

4. *VBHC application phase (post-intervention phase)*: the VBHC approach was applied in 2021. Outcomes were collected throughout and subsequently analyzed at the end of the intervention period. In terms of surgical standards, there were no major differences between this phase and the preintervention phase.

This study followed the SQUIRE 2.0 guidelines [18]. In-depth description of VBHC initiative development and staff training is reported in Supplementary material 1.

### Data reporting and statistical analysis

Potential sources of bias in cohort study were identified in selection and reporting bias. Selection biases were considered to have minimal impact in this cohort study, given the nature of EHR analysis (there was no change in EHR structure between 2019 and 2021), the study selection criteria, and the large population extracted. Reporting bias may have influenced the quality and completeness of entered data. For example, some data may be occasionally under-reported; however, this type of error is unlikely to have changed over time or have significant time trends, and is limited by the medical-legal requirements which mandate completeness of EHR documentation*.*

Variables were described by frequencies (percentage) or mean (SD) and median (IQR), as appropriate. Univariate associations were tested by chi-square test, and Mann–Whitney *U* test as appropriate, considering a threshold of 0.05 for statistical significance. All statistical analyses were performed using R software, version 4.2.1.

## Results

We included data from 11,013 hospital admissions, with 5242 patients admitted during the preintervention phase and 4480 patients admitted during the post-intervention phase.

Table 1 presents a comparison of patient demographics between those undergoing day surgery and those admitted for in-hospital stays. Day surgery accounted for 26.8% (2955 cases) of all surgical procedures. The patients’ mean age was slightly older than 60 years, and the age was generally lower in the day surgery cohort. In both 2019 and 2021, the proportion of patients with ASA class II disease was greatest. Higher-risk patients (ASA III and IV) were present in 23% of cases and were lower in the day surgery cohort (10.5%). The BMI was comparable between the subgroups and was in the range of normality for most patients.

**Table 1:** Patient characteristics and outcomes

	**In-hospital patients**^**a**^				**Day surgery patients**^**a**^			
**Variable**	Overall, (*N* = 8058)	2019 (*N* = 4804)	2021 (*N* = 3254)	*p* value^b^	Overall (*N* = 2955)	2019 (*N* = 1337)	2021 (*N* = 1618)	*p* value^b^
**Age (years)**	63.0 (51.0, 72.0)	62.0 (51.0, 72.0)	63.0 (51.0, 73.0)	0.03	57.0 (45.0, 67.5)	57.0 (45.0, 68.0)	57.0 (45.0, 67.0)	0.8
**ASA Class**								
** ASA 1**	1327.0 (18.6)	898 (19.8)	396 (18.7)	0.003	817 (37.3)	415 (35.1)	402 (40.0)	0.04
** ASA 2**	4177.0 (58.4)	2669 (59.6)	1221 (57.0)		1141 (52.1)	634 (53.5)	507 (50.5)	
** ASA 3/4**	1648.0 (23.0)	931 (20.6)	516 (24.3)		230 (10.5)	135 (11.4)	95 (9.5)	
**BMI**	25.2 (22.6, 28.1)	25.0 (22.6, 27.8)	25.3 (22.6, 28.4)	0.20	24.8 (22.4, 27.6)	24.9 (22.6, 27.7)	24.6 (22.3, 27.4)	0.3
**Cancelled surgery**	0.0 (0.0)	0.0 (0.0)	0.0 (0.0)	> 0.99	1.0 (0.0)	1 (0.1)	0 (0.0)	0.27
**Mortality**	27 (0.3)	16 (0.3)	11 (0.3)	> 0.99	0.0 (0.0)	0.0 (0.0)	0.0 (0.0)	> 0.99
**LOS (days)**	3.0 (2.0, 7.0)	3.0 (1.0, 7.0)	3.0 (2.0, 7.0)	0.01	NA	NA	NA	
**ICU admission**	409.0 (5.1)	244 (5.1)	165 (5.1)	> 0.99	0.0 (0.0)	0.0 (0.0)	0.0 (0.0)	> 0.99
**ICU stay (days)**	1.0 (1.0, 2.0)	1.0 (1.0, 2.0)	1.0 (1.0, 2.0)	0.90	NA	NA	NA	
**New hospitalization**	157.0 (1.9)	95.0 (2.0)	62.0 (1.9)	0.89	0.0 (0.0)	0.0 (0.0)	0.0 (0.0)	> 0.99
**In-**hosp preop consultancies	285.0 (3.5)	168.0 (3.5)	117.0 (3.6)	0.87	9.0 (0.3)	3.0 (0.2)	6.0 (0.4)	0.47

*LOS* Length of hospital stay, *BMI* Body mass index, *ICU* Intensive care unit, *ASA* American society of anaesthesiologists physical status classification

^a^Variables as median (IQR) or frequency (percentage)

^b^Chi-square test or Mann–Whitney *U* test

### Clinical results

The overall mortality was 0.2%, with no statistically significant difference between preintervention and post-intervention cohorts. The ICU admission rate was similar (4.0% preintervention vs. 3.4% in post-intervention cohort, *p* = 0.11), while the ICU length of stay did not differ, with a median of 1 day in both cohorts (*p* > 0.99).

The in-hospital preoperative consultancies, which were considered a proxy of inadequate evaluation during the preoperative clinical assessment, were similar before and after the intervention (2.8% vs 2.5%, *p* = 0.39), with a nonsignificant decrease from the preintervention to the post-intervention group.

**Fig. 3:**
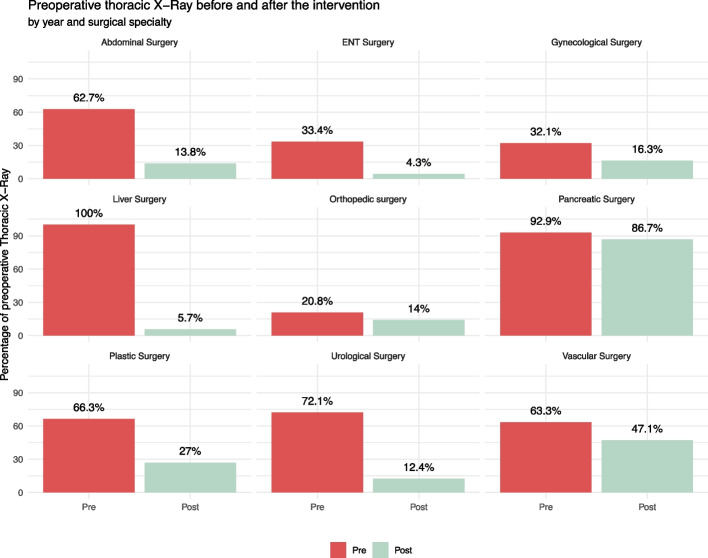
Thoracic X-ray before and after the intervention, broken down by year and surgical unit

There was a large decrease in the number of electrocardiograms, chest X-rays, and cardiology visits after the intervention compared to before the intervention, as reported in Table 2. Figure 3 shows how surgical units were affected by the reduction in preoperative chest X-rays after the introduction of the risk matrix.

**Table 2:** Variation in preoperative test and preoperative consultancies, before and after the intervention, and relative cost-analysis

**Preoperative test**	**2019** **(*N*, percent**^**a**^**)**	**2021** **(*N*, percent**^**a**^**)**	***p* value**^**b**^	**Delta volume**	**Delta cost (€)**	**Delta cost (%)**
EKG	6055 (107.2)	4508 (95.2)	< 0.001	− 1547	− 17,976	− 25.5%
Cardiology consult	3948 (69.9)	2538 (53.6)	< 0.001	− 1410	− 23,307	− 35.7%
Chest X-ray	3267 (57.9)	794 (16.8)	< 0.001	− 2473	− 34,474	− 75.7%
Preoperative anticoagulation clinic	975 (17.3)	770 (16.3)	0.18	− 205	− 3389	− 21.0%
Transthoracic cardiac ultrasonography	493 (8.7)	462 (9.8)	0.08	− 31	− 1340	− 6.3%
Spirometry	474 (8.4)	194 (4.1)	< 0.001	− 280	− 8134	− 59.1%
Pneumological consult	185 (3.3)	172 (3.6)	0.35	13	− 215	− 7.0%
Nutritional preoperative assessment	52 (0.9)	98 (2.1)	< 0.001	46	760	88.5%
Carotid duplex ultrasound	92 (1.6)	78 (1.6)	> 0.99	− 14	− 615	− 15.2%
Diabetic preoperative assessment	78 (1.4)	35 (0.7)	0.002	− 43	− 710	− 55.1%
TOTAL	15,619	9,649	–	− 5970	− 89,400	− 36.5%

EKG percentages sum up to more than 100%, as they could be repeated more than once (i.e., if an unplanned cardiology consult is requested)

*EKG* Electrocardiogram

^a^Percentages as a number of tests on number of preoperative pathways

^b^Chi-square test

The mean number of preoperative tests per hospital admission was significantly reduced from 6.15 exams per patient before the intervention to 5.32 exams per patient after the intervention (*p* < 0.001).

Supplementary materials 3 and 4 report the percentage variation in preoperative testing after the intervention, between day surgery and hospitalized patients.

### Cost results and other VBHC outcomes

The reduction in chest X-rays, cardiac consultations, and other preoperative tests as part of the intervention led to a decrease in overall costs. Table 2 shows that the reduction in the number of preoperative tests has resulted in an annual cost savings of nearly 90,000 €.

The decrease in preoperative test utilization not only cuts costs but also creates an opportunity to repurpose the freed diagnostic and human resources within the hospital. Through an internal analysis, we evaluated the potential cost savings from these released resources, taking into account the overall time saved by staff. Our analysis reveals that the value of these liberated resources is roughly equivalent to 95,000 €. Consequently, the total economic benefit can be estimated to be approximately 190,000 €.

The length of time spent by patients in the preoperative evaluation clinic was significantly lower after the intervention (373 vs. 290 min, *p* < 0.001). The value-time which represents the duration patients spend in preoperative clinics actually dedicated to medical activities and evaluations (excluding idle time), increased from 33 to 40%. All outcomes according to VBHC principles are reported in Table 3.

**Table 3:** Value-based outcomes in the whole cohort

Tier	Dimension	Outcome 2019 (*N*, percent)	Outcome 2021 (*N*, percent)	*p* value^c^
***Clinical outcome***	Mortality	16 (0.26)	11 (0.23)	0.75
***Process impacts***	ICU admissions	244 (4.0)	165 (3.4)	0.39
	Cancelled surgery (after hospital admission)	1.0 (0.02)	0.0 (0.00)	> 0.99
	Mean number of preoperative tests per patient	6.15 (2.50)^a^	5.32 (2.60)^a^	< 0.001
	Chest X-rays	3,267 (57.9)	794 (16.8)	< 0.001
	Preoperative cardiac consult	3,948 (69.9)	2,538 (53.6)	< 0.001
	Patient requested to access the preoperative clinic for more than 2 days	707 (11.7)	381 (8.2)	< 0.001
	Lead time in preoperative clinics (mins)	376 (129)^a^	295 (149)^a^	< 0.001
	Value time in preoperative clinics (mins)	109 (39)^a^	87 (31)^a^	< 0.001
	Value time (percent)	33% (19)	40 (29)	< 0.001
***Sustainability impacts***	Hospital length of stay (days)	3.0 (1.0, 7.0)^b^	3.0 (2.0, 7.0)^b^	0.01
	Medical controls requested in-hospital after the conclusion of preoperative assessment	171 (2.8)	123 (2.5)	0.34

^a^Mean (SD)

^b^Median (IQR). Percentages are calculated on the whole surgical population (day hospital and inpatients)

^c^Chi-square test or Mann–Whitney *U* test

## Discussion

In this pre-post quality improvement study, we demonstrated that an early personalized intervention to optimize preoperative assessment is safe, clinically feasible, and improves patient- and cost-relevant outcomes according to the VBHC value equation. A new tailored risk matrix was introduced at the first visit, for a total of 40 strata of risk despite a simple and rapid compilation backed by an automatic EHR procedure. The intervention resulted in reduced costs (lower number of preoperative tests and consultations), and time spent by patients in preoperative evaluation, without worsening clinical outcomes.

Before this VBHC initiative, the anaesthesiologist evaluated the patient at the end of the evaluation process which was standardized mainly by patient age. This causes a waste of resources and a potential delay in care, due to the request for additional tests and consult by the visiting anaesthesiologist at the end of the process. To limit this phenomenon, the hospital protocol before the VBHC initiative required several tests from baseline, leading to unnecessary testing and waste of resources [19, 20].

To avoid these limitations, we created a new risk matrix designed to route the patient at the beginning of the process. Anaesthesiologists and surgeons remain in charge of decision-making on preoperative evaluation (clearing patients for surgery), while delays, rescheduling, and unnecessary evaluations are reduced without an increase in costs, enhancing patient care and comfort or enhancing value from a multi-stakeholder perspective.

A few cases were reported by Mahajan et al. [21]: Through the establishment of a Centre for Perioperative Management and Medical Informatics, the Brigham and Women’s Hospital analyzed short- and long-term outcomes to improve perioperative patient safety, evaluating strategies to promote quality in perioperative care. In a study on patients undergoing joint replacement at Duke University (Durham, NC, USA), the authors found that the traditional pre-anesthesia evaluation model has minimal potential to influence the management of chronic comorbid medical conditions. They proposed screening patients early on for modifiable risk factors such as malnutrition, anemia, and diabetes to enroll them in dedicated preoperative clinics to improve outcomes and improve value in the process [22]. Our approach to preoperative management was broader than previous literature, encompassing the majority of surgical specialties. Patients were rerouted since the surgical visit to different preoperative pathways based on tailored risk strata, a process that was simplified by the hospital's electronic medical record. This strategy allows for increased value while preserving surgical safety and clinical outcomes. In fact, our strategy did not increase adverse events like death, ICU admission, hospital readmissions, or proxies of detrimental outcomes such as length of hospital or ICU stay. We also did not increase the use of adverse organizational metrics, such as last-minute surgical cancellations. In contrast, the reduced number of tests and services required during the preoperative period reduced hospital expenditures [23].

In particular, the number of preoperative tests and visits was reduced by 36.5%, and this -considering the cost of each test and procedure, resulted in a direct economic impact of approximately 90,000 € in savings in a single year. Here, we refer to the hospital cost related to these procedures, while the cost of the national health system and the cost of personal medical insurance are unquestionably greater. Considering that the new preadmission process did not increase clinical complications and that unnecessary testing can lead to false-positive findings, requiring further testing and expenses (also in terms of patients’ physical and psychological suffering), any strategy able to preserve outcomes by reducing evaluation and testing should be promoted. Moreover, the dramatic reduction in volumes for specific tests such as cardiac consults, X-rays, cardiac ultrasonography, and other visits permits the freeing of both human and equipment resources, which can then be reinvested in new medical tasks to generate additional value for patients and for the national healthcare system. The personalized preoperative risk matrix reduced the overall number of preoperative tests, but for high-risk patients, in which it increased the number of visits and tests, catering to their specific needs. The VBHC intervention aims for improved value equivalence, not just cost reduction, by providing proper medical care to groups that benefit more, such as doubling preoperative nutritional assessments in the post-intervention phase compared to baseline.

To increase the value of care in a VBHC framework, patient participation is essential [24]. To increase the benefit for patients and caregivers, we incorporated patients’ perspectives into our intervention and used a patient-centered approach. In our study, we were able to reduce the length of time patients spent in a preoperative evaluation clinic. Patients’ total time spent in the preoperative clinic significantly decreased by approximately 90 min after the intervention. Moreover, the time used for medical assessment increased by approximately 130% (from 33 to 40% of the total time), indicating that the intervention was able to reduce the downtime between visits and tests, through a reduction in the number and reorganization of the process.

Aligning the interests of patients, healthcare professionals, and institutions is mutually beneficial. The hospital’s cost savings could be reinvested in other areas, leading to a more evenly distributed improvement in patient care. Similarly, the time saved by healthcare providers may be invested in other clinical activities, additional quality improvement, or improved quality of life at no additional cost. To implement VBHC measures, reliable information technology, and a dedicated quality improvement unit are paramount. In this view, operational initiatives including tele-preoperative assessment, and technologies (i.e., videos, websites, and apps for patient education) should be promoted to improve patient engagement [25, 26] and reduce delays. In our experience, these technologies allow to increase patients’ education and improve patients’ safety and satisfaction and may be further used to improve value in healthcare at minimal additional costs [27].

### Limitations

Due to the high surgical volume and specific organizational characteristics, the external validity of this single-center study may be somewhat limited. However, the majority of the limitations that are common in preoperative evaluation clinics are diffuse across hospitals, and we believe that our strategy of early personalized preoperative evaluation pathways can be replicated by other hospitals to improve VBHC outcomes. Accordingly, we describe the initial experience of the application of this VBHC to another hospital with our organization in Supplementary material 5.

This was an observational before-after study, to measure the effect of a large organizational intervention. While the authors took all the possible precautions to minimize the risk of confounders, observational studies are susceptible to the risk of confounders, and the influence of some confounding variables may be difficult to quantify. We also acknowledge that this study may be underpowered to detect differences in major clinical outcomes, such as mortality and severe complications, which are rare in elective surgical populations. Moreover, due to the nature of the data extracted, we did not conduct a propensity score matching. Nonetheless, the comparison of characteristics between the groups before and after the intervention revealed minor and clinically negligible variations in baseline characteristics, supporting the validity of our approach.

Another limitation regards the measured compliance with the intervention. During the initial implementation of the preoperative pathway, we did not specifically collect data on staff compliance or attendance at educational interventions. After the VBCH initiative, while not all possible deviations from the new standard could be collected, we collect specific outcomes such as new requests for preoperative tests by physicians after patient evaluation during the preoperative clinic. These deviations, which were requested upon clinical examination by the physician, were less than 1% of the total number of chest X-rays and 8% of the total number of preoperative cardiology consults and are presented by surgery in Supplementary material 6.

The VBHC initiative began before the pandemic, but its deployment was delayed during the initial stages of the SARS-CoV-2 pandemic when hospital accessibility was limited for caregivers and families. Nonetheless, the pandemic was a catalyst for reorganization, as it increased healthcare providers' education and the rate of deployment of the risk matrix. The COVID-19 pandemic highlighted the significance of preserving the continuity of care, and this project helped to sustain this continuity during the third and fourth waves in the Lombardy region in 2021, as spending less time in the preoperative clinic was associated with a decreased risk of COVID-19 exposure from ambient air.

## Conclusions

In this before-and-after quality improvement study, we demonstrated that the application of VBHC principles to preoperative care through a structured organizational intervention is safe, clinically feasible, and can improve value in healthcare. With a general trend toward increased healthcare costs and decreased economic resources, the anaesthesiologist and hospital administrators should assume even greater responsibility for the perioperative process and promote a VBHC approach to enhance value in healthcare by aligning hospital interests with those of patients and society.

### Supplementary Information

**Additional file 1:** **Supplementary material 1.** Supplemental methods. **Supplementary material 2.** Algorithmic Flowchart of Medical Questionnaire decision process. **Supplementary material 3.** Bubble chart representing the use percentage variation in preoperative testing and clinics before and after the intervention, comparing Hospitalized and Day Surgery patients. **Supplementary material 4.** Spider plot on preoperative test and exam variations. **Supplementary material 5.** Initial experience of external validation in another hospital from our organization. **Supplementary material 6.** Compliance with preoperative pathway.

## Data Availability

The datasets used and/or analyzed during the current study are available from the corresponding author on reasonable request.

## Abbreviations

VBHC: Value-based healthcare
ICU: Intensive care unit
EHR: Electronic health record
ASA: American Society of Anesthesiologists
