# Correction: Value-based preoperative assessment in a large academic hospital

**DOI:** 10.1186/s44158-024-00184-0

**Published:** 2024-07-30

**Authors:** Maurizio Cecconi, Giulia Goretti, Andrea Pradella, Patrizia Meroni, Martina Pisarra, Guido Torzilli, Marco Montorsi, Antonino Spinelli, Alessandro Zerbi, Carlo Castoro, Paolo Casale, Efrem Civilini, Vittorio Quagliuolo, Marco Klinger, Giuseppe Spriano, Domenico Vitobello, Leonardo Maradei, Bernhard Reimers, Federico Piccioni, Maria Rosaria Martucci, Niccolò Stomeo, Elena Vanni, Marco Babbini, Roberta Monzani, Maria Rosaria Capogreco, Michele Lagioia, Massimiliano Greco

**Affiliations:** 1 Department of Biomedical Sciences, Humanitas University, Pieve Emanuele, Milan, Italy; 2 Department of Anaesthesiology and Intensive Care, IRCCS Humanitas Research Hospital, Rozzano, Milan, Italy; 3 IRCCS Humanitas Research Hospital, Rozzano, Milan, Italy; 4 Department of Economics, Management and Quantitative Methods, University of Milan, Milan, 20122 Italy; 5 Department of Surgery, IRCCS Humanitas Research Hospital, Rozzano, Milan, Italy; 6 Department of Cardiology, Humanitas Clinical and Research Center IRCCS, Milan, Italy

**Correction** **:** **J Anesth Analg Crit Care 4, 42 (2024)**

**https://doi.org/10.1186/s44158-024-00161-7**

In the original publication of this article [1] there was an incorrect author name. The incorrect and correct information is listed in this correction article. The original article has been updated.

Incorrect

Spinelli Antonino

Correct

Antonino Spinelli
