# Supplementary material for: Value-based preoperative assessment in a large academic hospital
Source: J Anesth Analg Crit Care. 2024 Jul 8;4:42. doi: 10.1186/s44158-024-00161-7 (PMC11232329; doi:10.1186/s44158-024-00161-7)
Supplement: Supplementary file 1 — Additional file 1: Supplementary material 1. Supplemental methods. Supplementary material 2. Algorithmic Flowchart of Medical Questionnaire decision process. Supplementary material 3. Bubble chart representing the use percentage variation in preoperative testing and clinics before and after the intervention, comparing Hospitalized and Day Surgery patients. Supplementary material 4. Spider plot on preoperative test and exam variations. Supplementary material 5. Initial experience of external validation in another hospital from our organization. Supplementary material 6. Compliance with preoperative pathway. [file 44158_2024_161_MOESM1_ESM.docx]

**Supplemental Material 1:**

**Supplemental methods**

*Intervention development*: the intervention included the following surgical units: general surgery, liver surgery, colorectal, upper gastrointestinal, pancreatic surgery, gynaecology, otorhinolaryngology, plastic surgery, urology, vascular surgery, and orthopaedic surgery.

Moving from previous initiatives focused on single pathways and processes, the hospital implemented a systematic Value Based Care Model, including the following steps.

1. *Evidence-based re-design of preoperative pathway.* This phase included the validation of clinical pathways in compliance through an evidence-based evaluation and was conducted by multidisciplinary teams and through structured meetings between clinicians.
2. *Organizational process*. After the revision of the clinical pathway, we designed the organizational process (including the operationalization of the clinical pathway), and designed a digital governance system with indicators and metrics.
3. *Business case*. This phase allowed the creation of the economic analysis (including costs and sustainability), the definition of the action plan, and the validation of a final dashboard with indicators on outcomes, process, quality of care and costs.

*Training and validation phase*: The entire year of 2020 was spent to training personnel, validating the intervention, and establishing the informatic support to automate the procedure. During the validation phase, the questionnaire was used and modified according to surgeons’, patients’ and anaesthesiologists’ feedback, and was maintained simple and quick in its development. The questionnaire can indeed be completed in a few minutes based on patients’ answers.

To exemplify, if the patient answers “no” to three initial screening questions, the questionnaire stops and the patient is directly classified as low medical risk. In contrast, the questionnaire continues, but “red/stopping” answers directly designate the patients at high-medical risk.

The medical risk questionnaire was maintained the same across all areas. In contrast, each IPUs classified the surgical interventions, ICD-9 code by ICD-9 code, in the three different surgical risks (low, medium, and high). The 11 IPUs' unique risk matrices were created by combining these two risk within each of the 11 IPUs. To facilitate this process, we were able to build an automated procedure directly into the EHR system. In this automated process, the clinicians follow the medical risk questionnaire directly on the EHR, and similarly the surgical risk is automatically extracted from the ICD9 code. In a few “clicks”, the EHR automatically generates the preoperative risk matrix and assign the patient to a category of risk and to the corresponding preoperative pathway.

**Supplemental material 2: Algorithmic Flowchart of Medical Questionnaire decision process**

**
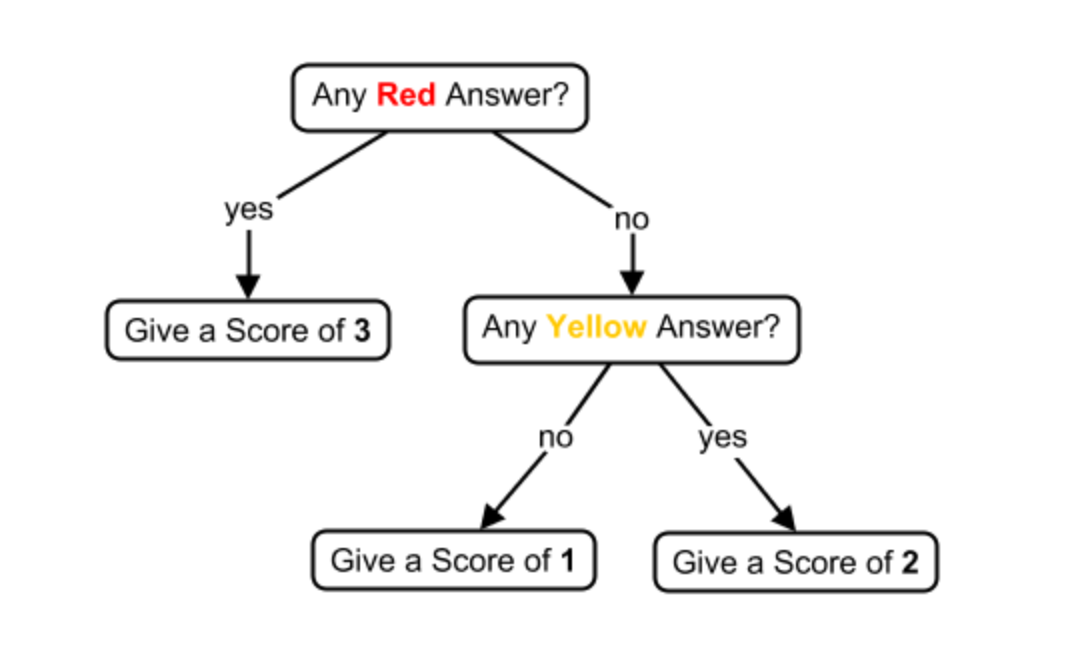
**

**Supplemental material 3: Bubble chart representing the use percentage variation in preoperative testing and clinics before and after the intervention, comparing Hospitalized and Day Surgery patients**

**
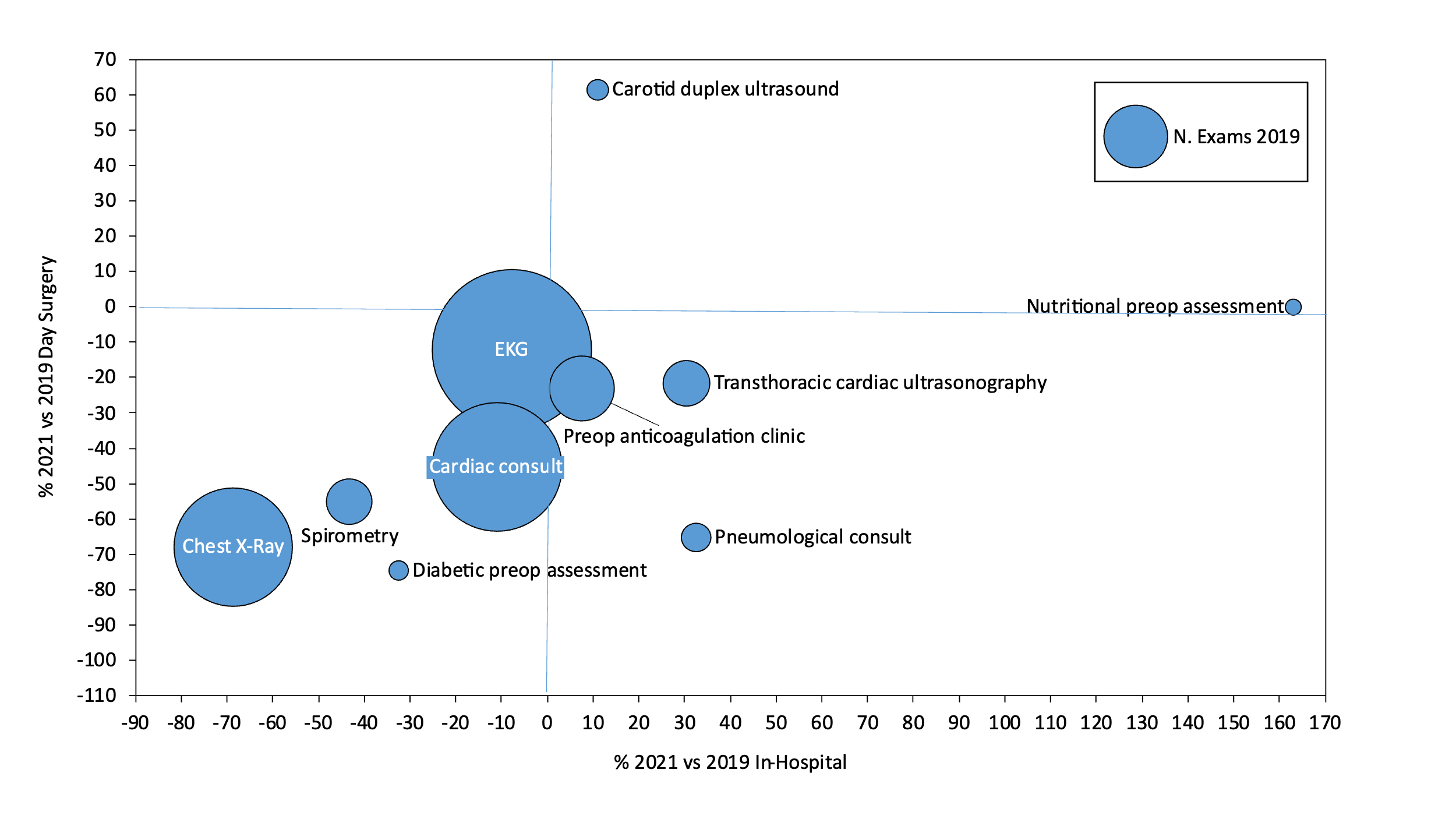
**

**Supplemental material 4: Spider plot on preoperative test and exam variations**

Figure A represents the percentage **reduction** in preoperative tests and visits before and after the intervention, in Hospitalized vs Day Surgery patients. Figure B represent the percentage **increase** **in** preoperative tests and visits. Figure C merges both in a single plot, representing the percentage variation (above and below 0%) of preoperative test and visits after the intervention.

**
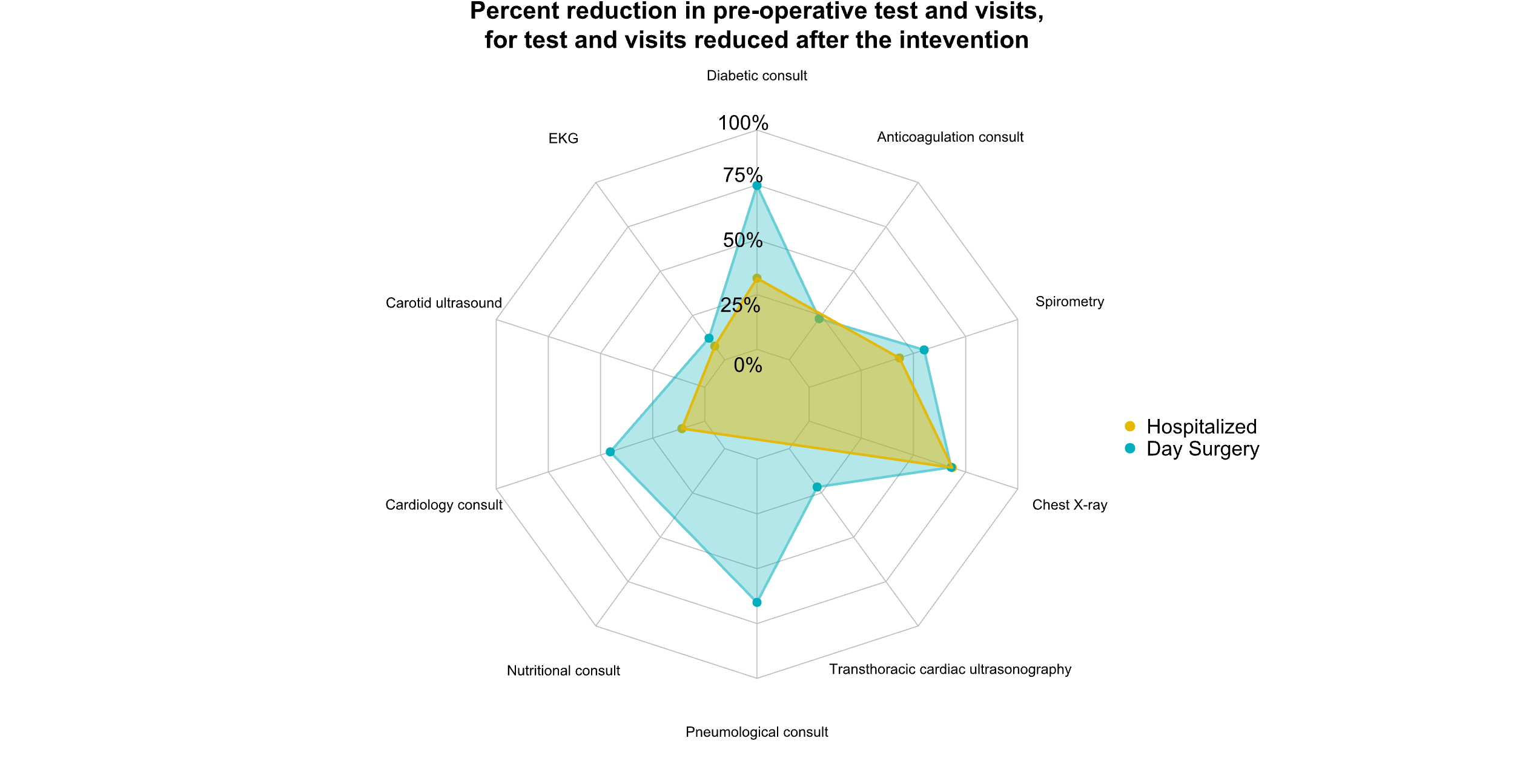
**

**
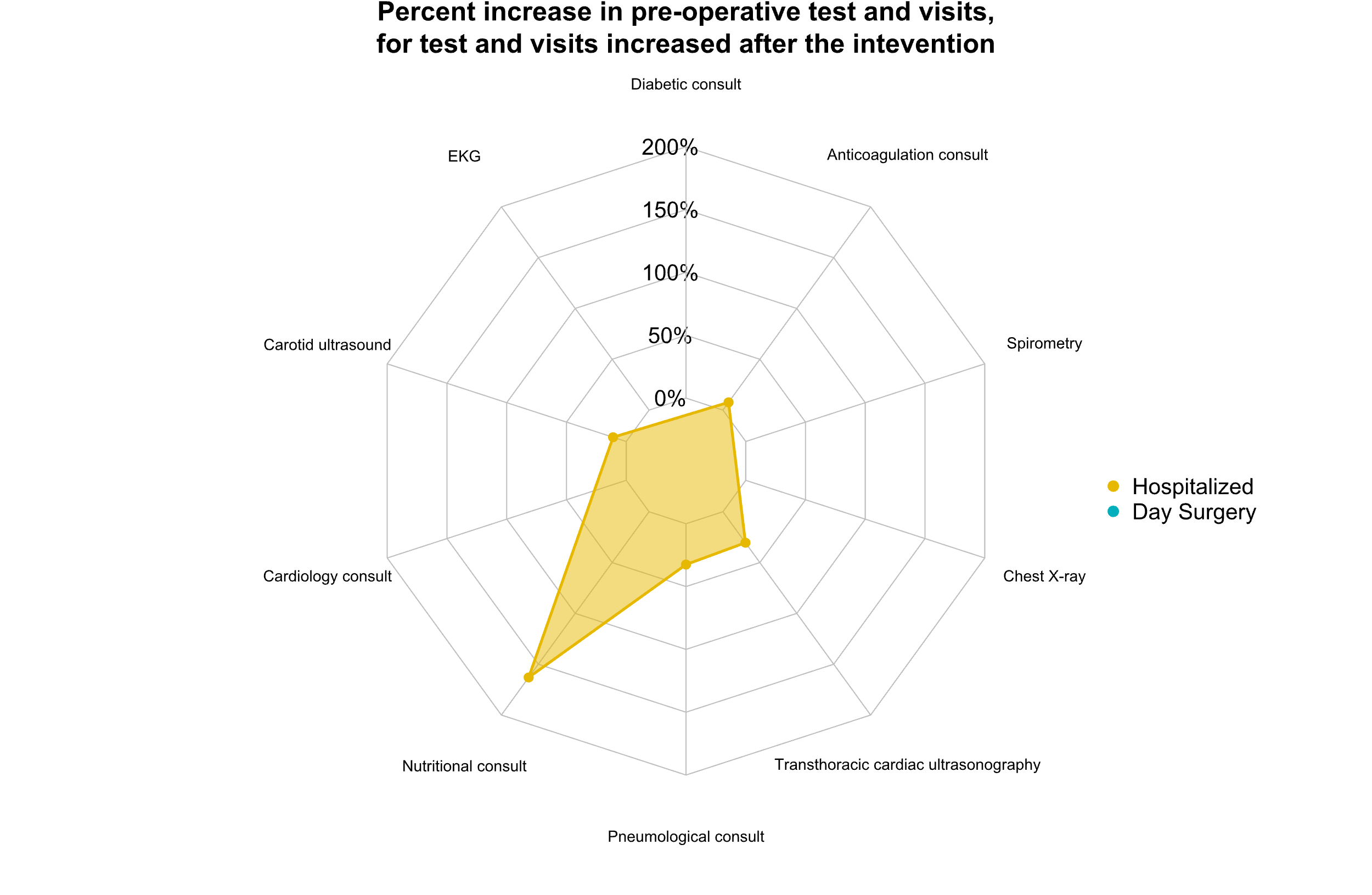
**

A

B

**
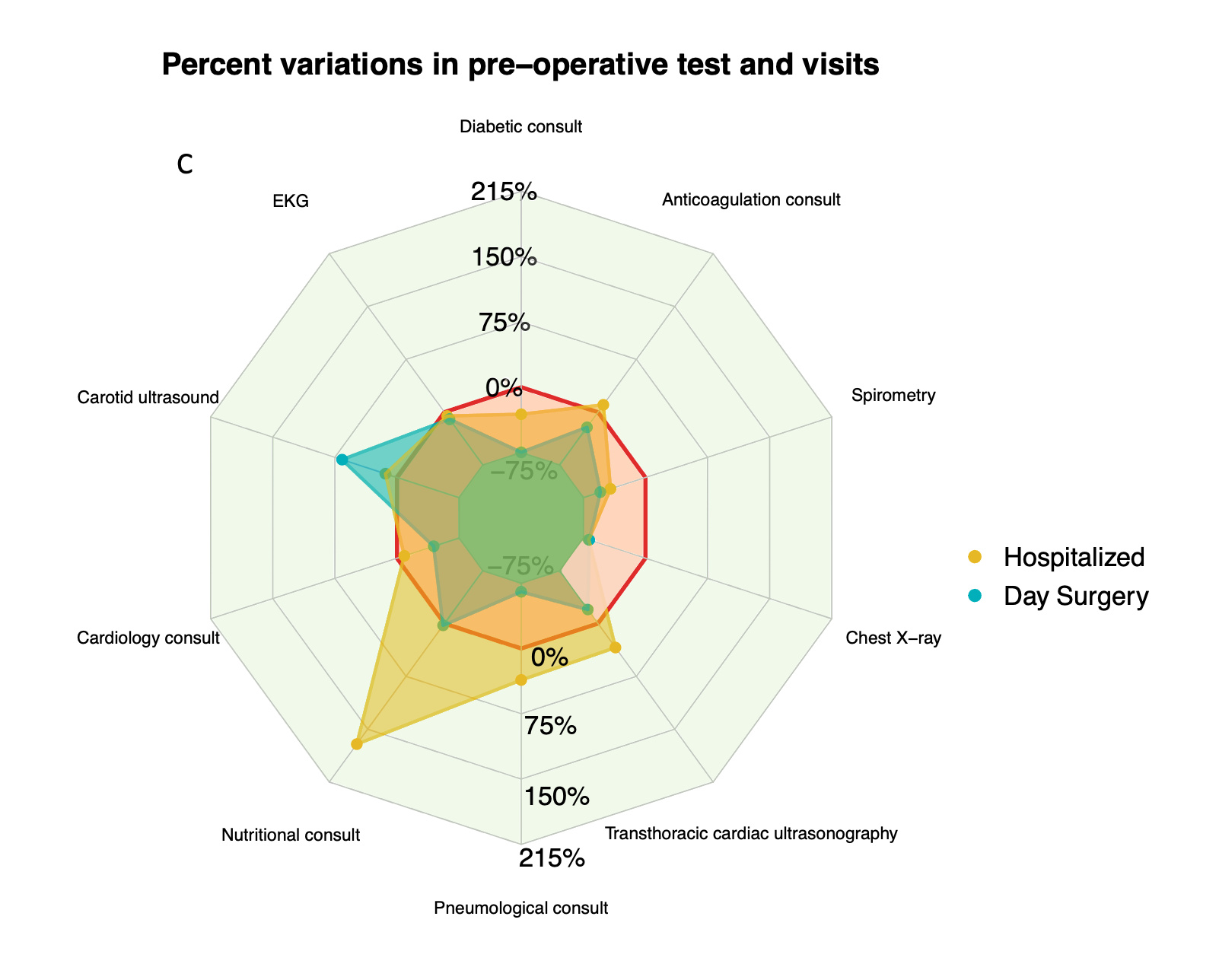
**

**Supplemental material 5:**

**Initial experience of external validation in another hospital from our organization.**

In past two years, we were able to transfer our VBHC strategy to a different hospital within our organization. The Humanitas Istituto Clinico Catanese, in Sicily, differs in terms of size (approximately 180 hospital beds compared to more than 800 in our study), surgical volume and surgical characteristics (mostly oncological patients). Moreover, it is situated in a different regional healthcare system (Sicilian regional healthcare system), which has some organizational differences compared to ours (to Lombardy Healthcare System). Even though we were unable yet to collect data from this secondary centre and evaluate efficacy in this context, the intervention was implemented without undue delay.

**Supplemental material 6: Compliance with preoperative pathway**

The table reports the percentage of adjunctive preoperative test requested by physician during preoperative consult, over the total number of the test in the preoperative pathway.

| Test | Abdominal surgery | Liver  surgery | Plastic  surgery | Vascular  surgery | Pancreatic  surgery | Gynecological surgery | ENT surgery | Orthopedic  surgery | Urological  surgery | Overall |
| --- | --- | --- | --- | --- | --- | --- | --- | --- | --- | --- |
| Chest X-ray | 1.8% | 1% | 0% | 1% | 1% | 1% | 1% | 1% | 2% | 1% |
| Preoperative cardiology consult | 6.8% | 9% | 7% | 11% | 9% | 6% | 8% | 7% | 12% | 8% |
